# Supplementary material for: A marmoset model for Mycobacterium avium complex pulmonary disease
Source: PLoS One. 2023 Mar 9;18(3):e0260563. doi: 10.1371/journal.pone.0260563 (PMC9997968; doi:10.1371/journal.pone.0260563)
Supplement: Attachment — Submitted filename: ACADEMIC EDITOR Response 11 27 2022.docx [file pone.0260563.s003.docx]

**11/27/2022**

ACADEMIC EDITOR: Though the authors have elaborately justified their views to reviewer comments, Several key points, related to limitations of the study, are missing in the revised manuscript itself. The authors should incorporate their response to reviewers into the discussion section that deals with limitations of the study. For example, the following should be mentioned:

**The inoculum of Mycobacterium avium complex was chosen based on an inoculation dose causing MAC disease in rhesus macaques (Am J Respir Cell Mol Biol; 54(2):170-176).**

**This study was not intended to investigate a dose-response threshold for establishing MAC infection in marmosets. That type of study would require a large number of animals and enormous expense.**

**Rather, the goal of this project was simply to determine if establishing pulmonary MAC infection was possible in the marmoset. Choosing an inoculation dose shown to cause pulmonary disease in another primate model appeared to offer the best chances of success with the least number of animals. It should be noted that we used the same inoculation dose of M. Abscessus in 3 marmosets and their chest radiographs, cytokines, and necropsy were completely normal over the 30-60 day period).**

**To the reviewer’s point about MAC inoculum in human disease, we submit that inoculum is unknown. Likely for humans, the size of the inoculum is not as important as the duration of the exposure.**

**Additionally, in humans, structural lung disease such as bronchiectasis appears to be necessary for MAC infection in the majority of cases so that it is not surprising in an animal with a normal tracheobronchial tree a relatively large inoculum would be required to establish infection.**

**It is also noteworthy that most current animal models for MAC disease require some systemic immune suppression or interruption of airway defenses or architecture. In that regard, the marmoset model offers the opportunity to assess MAC infection in an uncomplicated way.**

Similarly, the number of animals per timepoint (n=3-4) and usage of one sex (female) are confounding factors for proper statistical analysis**. Note: based on the reviewer’s comments, we discussed using the weekly cytokines for statistical analysis since this allowed for 5 -10 time points vs. a single time point for the BAL cytokines. This has been added to the final revision.**

***Revising the manuscript with these points would address the concern of the new reviewer comments posted below.***

**We are grateful to the Academic Editor for the advice and guidance. As suggested, we have added the following paragraph describing limitations of the study.**

***There are several limitations to this study. This study was not intended to investigate a dose-response threshold for establishing MAC infection in marmosets. That type of study would require a large number of animals and enormous expense. Rather, the goal of this project was simply to determine if establishing pulmonary MAC infection was possible in the marmoset. Choosing an inoculation dose shown to cause pulmonary disease in another primate model appeared to offer the best chances of success with the least number of animals, therefore, the inoculum of MAC was chosen based on an inoculation dose causing MAC disease in rhesus macaques (Winthrop K, et al. Am J Respir Cell Mol Biol. 2016;54(2):170-6). The MAC inoculum required to cause human disease is unknown. Likely for humans, the size of the inoculum is not as important as the duration of the exposure. Additionally, in humans, structural lung disease such as bronchiectasis appears to be necessary for MAC*** ***infection in the majority of cases so that it is not surprising in an animal with a normal tracheobronchial tree a relatively large inoculum would be required to establish infection. It is also noteworthy that most current animal models for MAC disease require some systemic immune suppression or interruption of airway defenses or architecture. In that regard, the marmoset model offers the opportunity to assess MAC infection in a less complicated way. The number of animals per time-point (n=3-4) and usage of one sex (female) are confounding factors for proper statistical analysis. Female marmosets were chosen because MAC disease in humans occurs primarily in females. Additionally, the study was only carried out at two time points (30 and 60 days). It is unclear, if the study had been extended to later time points, whether the MAC infection would completely resolve or evolve into a latent infection with the possibility of recurrence.***

==============================

Reviewer #3: Authors has to carried out a marmoset model of animal experiment to study the pathogenesis of nontuberculous mycobacterial disease. After infection, authors monitored several disease parameters including bacterial burden in several organs, cytokine levels and histopathology at 30 and 60 days of post infection. However, this study has to face the following concerns.
Major comments:
Even though all marmosets were infected with high dose of inoculum (10E8 of MAC) by endobronchial inoculation, infection was established in only 5 animals out 7 marmosets. This result indicate that more than 25% animals did not get infection. Has this study enough statistical power to bring any conclusion? **As pointed out in the revised manuscript, two animals did not receive the full inoculum. The anesthesiologist was told to pull the scope back an inch since the scope was sitting on the carina and he removed the bronchoscope above the vocal cords and no MAC was instilled. A second animal had the isoflurane stopped early and the marmoset started coughing and vomited. Unlike the other animals, this marmoset had no crackles in the right lung (that was consistently noted in other marmosets). Finally, the only published non-human primate study had only 3 animals tested with 1 of 3 developing histology consistent with a granulomatous disease (see below)**

**.**

***In the rhesus macaque manuscript (******Winthrop K, et al. Am J Respir Cell Mol Biol. 2016;54(2):170-6) challenged 3 animals with Mycobacterium avium complex (MAC) and only one animal (33%) developed pulmonary MAC infection. We consider the successful infection of 5 animals to be remarkable and unprecedented. There are no similar data in non-human primates. Given there were technical problems with the MAC inoculation of the two uninfected animals (who did not receive the full inoculation) we are confident in the validity of the results from the infected animals. We also gave the same inoculum of M. Abscessus to three marmosets and were unable to induce any histologic or cytokine changes. This strongly suggests that the pathogenesis of non-tuberculous mycobacterial disease is dependent on both the virulence of the organism and the dose of inoculum delivered to the lung.***

The authors concluded that this study would be useful to recapitulate M. avium complex lung infection in humans. However, proper standardization of inoculum should be carried out to establish infection in all infected marmosets with minimum standard deviation.

***We agree with the reviewer that a larger, dose-response study is both desirable and necessary. That type of study would require a large number of animals and would be prohibitively expensive without major external funding. As we discussed in our initial reviewer responses, the goal of this study was to demonstrate that the marmoset offers a non-human primate model for studying MAC infection. We believe the marmoset model is the closest to recapitulation human MAC disease so far describe. Hopefully, with publication of this data, we might successfully acquire funding to pursue the more rigorous study suggested by the reviewer.***

***Based on the reviewers’ comments, we had the statistician at National Jewish review our data and create a statistical analysis of cytokine changes from baseline (pre-inoculation) to the time of necropsy. This has been added to the Result Section and Table 2 and Figure 4 added to the final manuscript. We appreciate each of the three reviewers’ comments and feel the changes to the final manuscript have improved it significantly.***
